# Lactate trajectories and outcomes in patients with sepsis in the intensive care unit: group-based trajectory modeling

**DOI:** 10.3389/fpubh.2025.1610220

**Published:** 2025-06-16

**Authors:** Yu Wei, Jinqiang Zhuang, Jiaqi Li, Zixuan Wang, Jing Wang, Xiaojie Zhang, Junling Leng

**Affiliations:** ^1^Emergency Department, The Affiliated Hospital of Yangzhou University, Yangzhou University, Yangzhou, China; ^2^Medical College of Yangzhou University, Yangzhou, China; ^3^Emergency Intensive Care Unit (EICU), The Affiliated Hospital of Yangzhou University, Yangzhou, China; ^4^School of Nursing and·School of Public Health, Yangzhou University, Yangzhou, China

**Keywords:** sepsis, lactate, group-based trajectory modeling, survival analysis, subphenotype

## Abstract

**Background:**

Sepsis is highly heterogeneous. Therefore, identifying biomarkers that can stratify patients with sepsis into more homogeneous cohorts to develop individualized treatment and care measures for patients and carry out early intervention to reduce the risk of death and improve the prognosis of patients has become a current research hotspot.

**Methods:**

Using the MIMIC-IV database, we analyzed data from 1,575 adult patients with sepsis. Serum lactate levels were measured once daily for 5 consecutive days after admission. The GBTM model was used to stratify the risk of sepsis and explore the relationships between different lactate trajectories and 28-day mortality in septic patients.

**Results:**

We report a new method for identifying subphenotypes of sepsis patients based on lactate trajectories. Through group-based trajectory modeling, we identified and validated five groups of sepsis patients with different lactate trajectories, namely, “Low-stable group,” “low-slowly declining group,” “high-rapidly decline group,” “Moderate-slow declining group,” and “high-slow decline group.” The relationships between sepsis patients with different lactate trajectories and 28-day mortality were explored. Among them, patients with a “Low-stable group” had the lowest in-hospital 28-day mortality. Patients with a “high-slow decline group” had the highest 28-day mortality.

**Conclusion:**

In this study, different subtypes of sepsis were successfully identified by analyzing lactate trajectories. Combined with the dynamic changes in lactate levels, the GBTM model was used to stratify patients according to their risk of sepsis. This model provides a theoretical basis for clinicians to evaluate the prognosis of patients using the lactate change trajectory.

## 1 Introduction

Sepsis refers to life-threatening organ dysfunction caused by a dysregulated host response to infection. Owing to its high mortality and morbidity rates and large economic burden, sepsis has become a public health issue (1). Rudd et al. recently reported 48.9 million sepsis cases and 11 million sepsis-related deaths (2) in 2017. More than 30 million hospital-treated sepsis cases are estimated to occur globally each year, with 5.3 million patients dying from sepsis, according to a systematic review published in 2016 (3) based on studies from high-income countries. China, the most populous country in the world, has an incidence of sepsis in adults of 236/100,000 people and a related death rate of 67/100,000 people (sepsis-related mortality rate of 32.0%). Specifically, approximately 2,487,949 patients are diagnosed with sepsis, and 700,437 patients die of sepsis (4) in China every year. In addition to domestic studies, the Institute for Health Metrics and Evaluation (IHME) of the University of Washington has published the global burden of disease of sepsis (GBD). The GBD (2) study revealed that there were 2,931,827 sepsis patients and 709,315 sepsis-related deaths in China in 2017, with a standardized sepsis incidence of 214.8 per 100,000 people and a mortality rate of 43.3 per 100,000 people. Sepsis is highly heterogeneous, and there are significant differences in the immune response, clinical manifestations, and prognosis between different patients. Therefore, biomarkers can be used to stratify sepsis patients for a more homogenous queue; thus, individualized treatment and nursing measures for patients, early intervention, reducing the risk of death, improving the prognosis, and achieving better treatment effects have become popular research topics.

Blood lactic acid, an intermediate product of circulation metabolism in the body, is produced by brain tissue, red blood cells, and striated muscle. The concentration of lactic acid in the body is mainly determined by the production rate and metabolic rate of the kidneys and liver. In some special cases (such as respiratory and circulatory failure), tissue cell edema, hypoxia, and hypoxia can lead to increased blood lactic acid levels in the body. In addition, in the circulation of glucose metabolism in the body, rapid glycolysis, dehydration, and strenuous exercise can also lead to an increase in blood lactate. Elevated blood lactate is considered a reliable marker (5–9) of disease severity and mortality, and increasing evidence indicates that lactate measurement is a valuable and direct indicator for the early identification of severe sepsis and is associated with poor prognosis. Although studies have investigated the relationship between sepsis and lactate levels, at present, no further studies have evaluated the prognosis of sepsis patients based on the value and change trend of lactate. In this study, we measured lactate values at different times, identified sepsis subphenotypes via group-based trajectory modeling, analyzed the characteristics of sepsis subphenotypes, and stratified the patients according to the prognostic risk of sepsis to use the lactate trajectory to evaluate the prognosis of sepsis.

Group-based trajectory modeling is a method used to analyze individual behavior changes during a specific period of time. The GBTM model identifies different trajectories, putting the individual on a similar trajectory in the group. Such models are particularly well suited to handle longitudinal data, where individuals are observed to trend over a phenomenon. It is based on the application of finite mixture models that use groups of trajectories as statistical tools for approximating the unknown trajectories of population members. A trajectory group is defined as a cluster of individuals who follow similar trajectories in terms of outcomes over time. GBTM models are widely used in sociology, psychology, and public health research. For example, in public health research, group-based trajectory modeling (GBTM) has been widely utilized to characterize longitudinal trends in disease incidence (10), symptom trajectories (e.g., pain, depression, and anxiety), and scale-based outcomes (11, 12). In this study, GBTM was selected for its methodological advantages, including straightforward model simplicity, robust interpretability, and the ability to generate clinically actionable classifications. Specifically, GBTM identifies subgroups of patients with homogeneous dynamic trajectories, which aligns with the heterogeneous treatment response hypothesis. This explicit stratification facilitates precision medicine by guiding tailored therapeutic strategies and optimizing resource allocation for distinct patient subpopulations.

## 2 Materials and methods

### 2.1 Subjects

This study was a retrospective, observational study. Based on the MIMIC-IV database, the data used in this study were obtained from the MIMIC-IV database, which contains electronic health record (EHR) data from intensive care unit (ICU) patients and is provided by Beth Israel Deaconess Medical Center (BIDMC) in Boston, Massachusetts, United States. The MIMIC-IV database contains detailed clinical data, including physiological parameters of patients, laboratory test results, and drug use records, and has been widely used in clinical research. The study analyzed patient data from 2008 to 2022 and included data on all intensive care patients during this period. The inclusion criteria for this study were as follows: (1) age ≥18 years; (2) sepsis diagnosis based on Sepsis-3 criteria (1), defined as documented or suspected infection accompanied by an acute increase in the Sequential Organ Failure Assessment (SOFA) score of ≥2 points; (3) first-time admission to the intensive care unit (ICU) with a minimum ICU stay of 5 days; (4) serial lactate measurements obtained every 24 h during the first 5 days following ICU admission. The exclusion criteria were as follows: (1) age less than 18 years, (2) admission time less than 5 days, (3) fewer than five lactic acid tests were performed in the first 5 days after admission, and (4) repeated admission.

### 2.2 Data collection

The following basic information of the patients with sepsis were extracted: sex, age, length of ICU stay, 28-day survival outcome, APSIII score, SOFA score, vasoactive drugs, sedative and analgesic use, creatinine, alanine aminotransferase, aspartate aminotransferase, red blood cell count, white blood cell count, hemoglobin, platelets, bilirubin total, heart rate, systolic blood pressure, diastolic blood pressure, respiratory rate, complications, All the above laboratory variables were the most severe values within 24 h after the diagnosis of sepsis. The total amount of daily fluid infusion was continuously monitored and recorded for 5 days after admission, and We collected the lactate data for patients within the first 5 days after ICU admission. The lactate values of day 1, day 2, day 3, day 4, and day 5 were collected and labeled as Lac1, Lac2, Lac3, Lac4, and Lac5, respectively. The endpoint was 28-day mortality.

### 2.3 Missing values

Variables with > 30% missing values were excluded. Albumin, C-reactive protein, and fibrinogen all had > 30% missing values and were therefore excluded. For variables with missing values of less than 30%, we used the multiple imputation (MI) method using the mice package in R to address missing data. Specifically, we utilized the Classification and Regression Trees (CART) algorithm as the imputation method, which predicts missing values based on decision tree models. The CART method is capable of capturing complex non-linear relationships and applies to various types of variables. We generated five imputed datasets (m = 5) and applied Rubin’s rules to combine the imputation results, thereby reducing the uncertainty and enhancing the robustness of the statistical analysis.

### 2.4 Statistical methods

Continuous data with a normal distribution are expressed as the mean ± standard deviation (SD), and continuous data with a skewed distribution are expressed as M (Q1, Q3). The rank sum test was used for multiple groups, and analysis of variance was used for comparisons among multiple groups. The classified data are expressed as n (%), and the chi-square test or Fisher’s exact probability method was used. A Kaplan–Meier curve was used for survival analysis, and a Cox regression model was used to study the survival time of patients and the predictive factors related to survival time. For all comparisons, a *p*-value of less than 0.05 was considered statistically significant.

The R language was used for group-based trajectory modeling. To select the optimal number of categories, we built models with 1–5 categories. According to the Bayesian information criterion (BIC) and the Akaike information criterion, the closer the BIC and AIC are to 0, the better the model fitting effect is. AvePP>0.7 indicates that the model is acceptable. In addition, the goodness-of-fit of the model was ensured by verifying that the average posterior probability of all classification members was ≥70%. Finally, we considered the clinical interpretability of the model.

## 3 Results

### 3.1 Basic patient characteristics

The mean age of the study subjects was (62.37 ± 15.97) years. The proportion of male patients was 59.17%; 1,071 patients survived, and 504 patients died. The overall 28-day mortality rate was 32.0%, the SOFA score was 4.00 (3.00, 7.00), and the duration of ICU stay was 12.52 (8.64, 20.10) days.

### 3.2 Value of lactic acid in the prognosis of patients with sepsis

**Figure 1:**
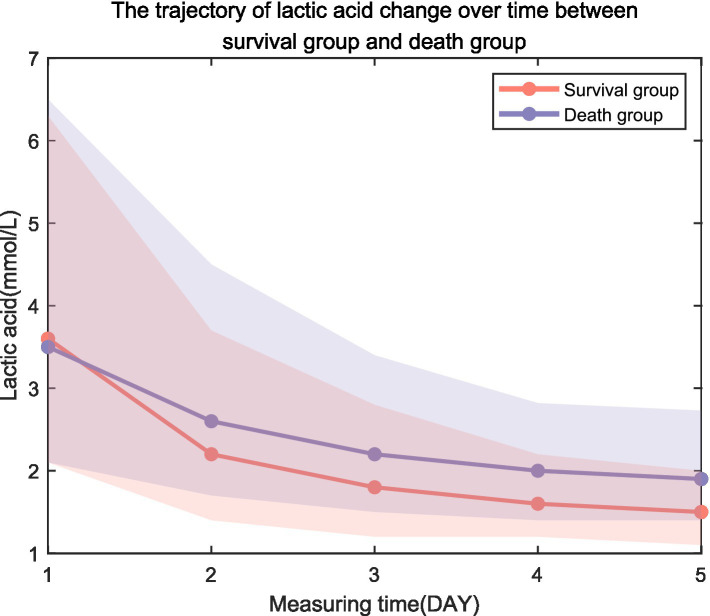
Trajectory of lactic acid change over time between the survival group and the death group.

The lactate trajectories of the surviving group and the non-surviving group tended to decrease (Figure 1). LAC1, LAC2, LAC3, LAC4, and LAC5 were used to evaluate their ability to predict sepsis prognosis, and the results revealed that the lactic acid values of the death group were greater than those of the survival group (*p* < 0.05; Table 1).

**Table 1:** Comparison of lifestyles at different times of hospital admission in the survivor and death groups.

Variables	Total (*n* = 1,575)	Survive (*n* = 1,071)	Death (*n* = 504)	Statistic	*p*
Lactate 1(Q₁, Q₃)	3.60 (2.10, 6.40)	3.60 (2.10, 6.30)	3.50 (2.10, 6.50)	Z = −0.41	0.681
Lactate 2(Q₁, Q₃)	2.30 (1.50, 4.00)	2.20 (1.40, 3.70)	2.60 (1.70, 4.50)	Z = −5.21	<0.001
Lactate 3(Q₁, Q₃)	1.90 (1.30, 3.00)	1.80 (1.20, 2.80)	2.20 (1.50, 3.40)	Z = −6.30	<0.001
Lactate 4(Q₁, Q₃)	1.70 (1.20, 2.40)	1.60 (1.20, 2.20)	2.00 (1.40, 2.82)	Z = −8.50	<0.001
Lactate 5(Q₁, Q₃)	1.60 (1.20, 2.20)	1.50 (1.10, 2.00)	1.90 (1.40, 2.73)	Z = −10.70	<0.001

### 3.3 Group trajectory modeling

Group trajectory modeling was performed on 1,575 patients with sepsis in this cohort. The results revealed that as the number of trajectory groups increased from 1 to 5, the AIC and BIC decreased. When the number of trajectory groups was 5, the Avepp values were 0.92, 0.90, 0.89, 0.90, and 0.95, respectively, which were greater than the empirical standard of 0.70. The fit of the model was considered good (Table 2). The number of trajectory groups of 5 was determined as the final result.

**Table 2:** Group-based trajectory modeling for choosing the best number of phenotypes.

model	G1	G2	G3	G4	G5	AIC	BIC	CAIC	SSBIC	HQIC
traj_1	1.0	N/A	N/A	N/A	N/A	36733.10	36767.95	36772.95	36752.06	36745.04
traj_2	0.9742605	0.9747231	N/A	N/A	N/A	30024.58	30101.26	30112.26	30066.31	30050.85
traj_3	0.9546785	0.9333887	0.9486020	N/A	N/A	28079.92	28191.46	28207.46	28140.61	28118.13
traj_4	0.9325649	0.9323963	0.9476263	0.9124386	N/A	27123.21	27283.55	27306.55	27210.46	27178.13
traj_5	0.9203401	0.8950656	0.8921665	0.8999539	0.947905	26584.32	26779.52	26807.52	26690.54	26651.19

**Figure 2:**
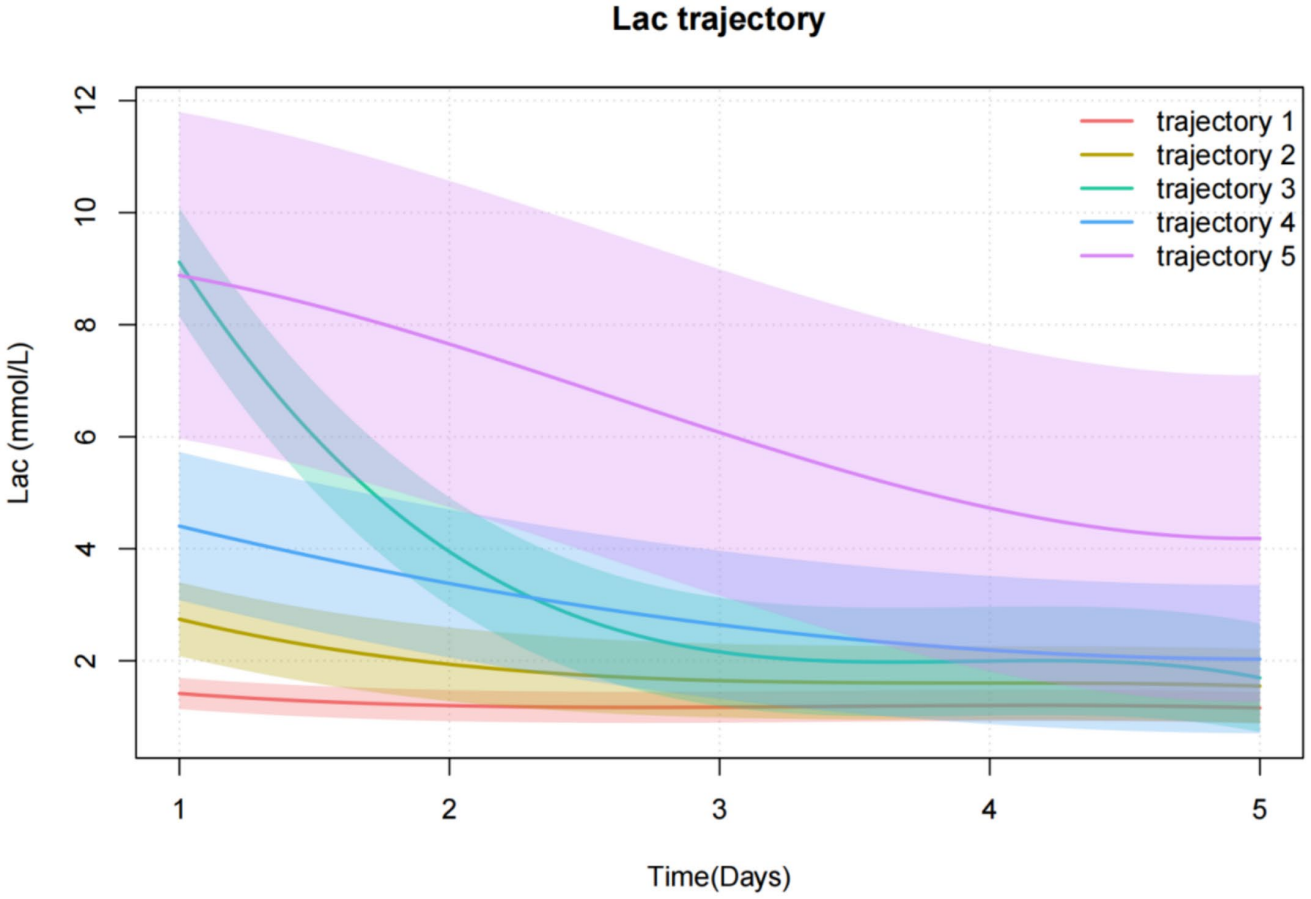
Lactate trajectories based on GBTM.

Trajectory 1 (*n* = 246, 15.6%) was characterized by the lactate levels were consistently low and did not fluctuate much, which was defined as a “Low-stable group.” Trajectory 2 (*n* = 464, 29.5%) was characterized by the lactate levels were low and slowly decreased, which was defined as the “low-slowly declining group.” Trajectory 3 (*n* = 225, 14.3%) was characterized by lactate levels that were high initially and decreased rapidly. It was defined as a “high-rapidly decline group.” Trajectory 4 (*n* = 435, 27.6%) was characterized by the lactate levels that were moderate at first and decreased slowly over time, which was defined as a “Moderate-slow declining group.” Trajectory 5 (*n* = 205, 13.0%) was characterized by the lactate levels that were initially extremely high and decreased slowly, which was defined as a “high-slow decline group” (Figure 2).

### 3.4 Comparison of clinical data between trajectory groups

#### 3.4.1 Comparison of demographics and complications

Among 1,575 participants, the mean (±SD) admission age was 62.4 ± 16.0 years, with no significant differences across groups (range: 61.8 ± 16.0 to 63.1 ± 16.8; *p* = 0.81). Overall, 40.8% of the participants were female, with comparable sex distribution among groups (*p* = 0.42). Chronic pulmonary disease prevalence varied (*p* = 0.02), with the highest proportion in “Low-stable group” (32.1%) and the lowest in “high-slow decline group” (21.5%). A trend toward differential malignant cancer prevalence was observed (overall 10.5%; range: 6.5% [“Low-stable group”] to 14.6% [“high-slow decline group”]; *p* = 0.05). No significant between-group differences were detected for congestive heart failure (*p* = 0.20), cerebrovascular disease (*p* = 0.20), diabetes (*p* = 0.33), renal disease (*p* = 0.58), or metastatic solid tumor (Table 3).

**Table 3:** Comparison of demographics and comorbidities.

Variables	Total (*n* = 1,575)	Trajectory 1 (*n* = 246)	Trajectory 2 (*n* = 464)	Trajectory 3 (*n* = 225)	Trajectory 4 (*n* = 435)	Trajectory 5 (*n* = 205)	*p*
Admission Age, Mean ± SD	62.37 ± 15.97	62.43 ± 15.75	62.80 ± 15.73	63.07 ± 16.77	61.76 ± 15.96	61.86 ± 15.95	0.806
Gender, n(%)							0.420
F	643 (40.83)	104 (42.28)	189 (40.73)	80 (35.56)	179 (41.15)	91 (44.39)	
M	932 (59.17)	142 (57.72)	275 (59.27)	145 (64.44)	256 (58.85)	114 (55.61)	
Congestive Heart Failure, n(%)							0.202
No	1,037 (65.84)	152 (61.79)	298 (64.22)	153 (68.00)	303 (69.66)	131 (63.90)	
Yes	538 (34.16)	94 (38.21)	166 (35.78)	72 (32.00)	132 (30.34)	74 (36.10)	
Cerebrovascular Disease, n(%)							0.195
No	1,340 (85.08)	198 (80.49)	395 (85.13)	194 (86.22)	372 (85.52)	181 (88.29)	
Yes	235 (14.92)	48 (19.51)	69 (14.87)	31 (13.78)	63 (14.48)	24 (11.71)	
Chronic Pulmonary Disease, n(%)							0.021
No	1,144 (72.63)	167 (67.89)	323 (69.61)	175 (77.78)	318 (73.10)	161 (78.54)	
Yes	431 (27.37)	79 (32.11)	141 (30.39)	50 (22.22)	117 (26.90)	44 (21.46)	
Diabetes, n(%)							0.325
No	1,075 (68.25)	164 (66.67)	313 (67.46)	167 (74.22)	290 (66.67)	141 (68.78)	
Yes	500 (31.75)	82 (33.33)	151 (32.54)	58 (25.78)	145 (33.33)	64 (31.22)	
Renal Disease, n(%)							0.582
No	1,194 (75.81)	180 (73.17)	346 (74.57)	178 (79.11)	333 (76.55)	157 (76.59)	
Yes	381 (24.19)	66 (26.83)	118 (25.43)	47 (20.89)	102 (23.45)	48 (23.41)	
Malignant Cancer, n(%)							0.050
No	1,409 (89.46)	230 (93.50)	416 (89.66)	205 (91.11)	383 (88.05)	175 (85.37)	
Yes	166 (10.54)	16 (6.50)	48 (10.34)	20 (8.89)	52 (11.95)	30 (14.63)	
Metastatic Solid Tumor, n(%)							0.333
No	1,518 (96.38)	241 (97.97)	443 (95.47)	220 (97.78)	418 (96.09)	196 (95.61)	
Yes	57 (3.62)	5 (2.03)	21 (4.53)	5 (2.22)	17 (3.91)	9 (4.39)	

#### 3.4.2 Comparison of physiological characteristics

Among 1,575 participants, the cohort was stratified into five groups (“Low-stable group”: *n* = 246 [15.6%]; “low-slowly declining group”: *n* = 464 [29.5%]; “high-rapidly decline group”: *n* = 225 [14.3%]; “Moderate-slow declining group”: *n* = 435 [27.6%]; and “high-slow decline group”: *n* = 205 [13.0%]). Significant differences were observed across the groups for most clinical and laboratory parameters. Hematologic and Biochemical Markers: Hemoglobin levels varied (mean 11.35 ± 2.32; “high-rapidly decline group”: 11.66 ± 2.11 vs. “Low-stable group”: 10.98 ± 2.25; *p* = 0.005). Platelet counts were highest in the “Low-stable group” (median 214.00 [IQR 165.00–302.75]) and lowest in the “high-slow decline group” (164.00 [118.00–230.00]; *p* < 0.001). Total bilirubin was elevated in the “high-slow decline group” (median 2.10 [0.90–5.30]) compared with the “Low-stable group” (0.70 [0.40–1.40]; *p* < 0.001). Organ Dysfunction Scores: SOFA scores differed significantly (overall median 4.00 [3.00–7.00]; “Low-stable group”: 3.00 [2.00–5.00] vs. “high-slow decline group”: 5.00 [3.00–8.00]; *p* < 0.001). APSIII scores increased progressively across groups (“Low-stable group”: 56.00 [42.25–73.75]; “high-slow decline group”: 82.00 [63.00–99.00]; *p* < 0.001). Vital and Metabolic Parameters: Heart rate was highest in the “high-slow decline group” (mean 119.34 ± 24.27 bpm) and lowest in the “Low-stable group” (105.57 ± 22.12 bpm; *p* < 0.001). Respiratory rate (median 30.00 [25.25–34.00]; *p* = 0.343) and diastolic blood pressure (median 81.00 [69.00–96.00]; *p* = 0.605) did not differ significantly. Liver Function Tests: ALT and AST levels were markedly higher in groups 3 and 5 (ALT: “high-slow decline group,” 107.00 [36.00–865.00]; AST: “high-slow decline group,” 241.00 [69.00–2501.00]) vs. “Low-stable group” (ALT: 32.00 [18.00–68.75]; AST: 51.50 [29.00–132.75]; both *p* < 0.001; Table 4).

**Table 4:** Comparison of physiological characteristics.

Variables	Total (*n* = 1,575)	Trajectory 1 (*n* = 246)	Trajectory 2 (*n* = 464)	Trajectory 3 (*n* = 225)	Trajectory 4 (*n* = 435)	Trajectory 5 (*n* = 205)	*p*
Rbc, Mean ± SD	3.78 ± 0.82	3.72 ± 0.74	3.73 ± 0.81	3.90 ± 0.72	3.82 ± 0.90	3.74 ± 0.86	0.054
Hemoglobin, Mean ± SD	11.35 ± 2.32	10.98 ± 2.25	11.23 ± 2.27	11.66 ± 2.11	11.55 ± 2.52	11.28 ± 2.25	0.005
Heart Rate, Mean ± SD	114.75 ± 23.72	105.57 ± 22.12	114.53 ± 23.45	118.20 ± 22.05	116.23 ± 24.15	119.34 ± 24.27	<0.001
Sofa Score, M (Q₁, Q₃)	4.00 (3.00, 7.00)	3.00 (2.00, 5.00)	4.00 (3.00, 6.00)	5.00 (3.00, 8.00)	5.00 (3.00, 7.00)	5.00 (3.00, 8.00)	<0.001
APSIII, M (Q₁, Q₃)	68.00 (53.00, 86.00)	56.00 (42.25, 73.75)	65.00 (52.00, 84.00)	74.00 (57.00, 95.00)	69.00 (54.00, 87.00)	82.00 (63.00, 99.00)	<0.001
Creatinine, M (Q₁, Q₃)	1.70 (1.10, 2.90)	1.30 (0.90, 2.50)	1.65 (1.00, 2.90)	2.00 (1.40, 2.80)	1.70 (1.10, 2.80)	2.20 (1.40, 3.20)	<0.001
Alt, M (Q₁, Q₃)	49.00 (23.00, 156.00)	32.00 (18.00, 68.75)	36.50 (21.00, 101.75)	101.00 (37.00, 442.00)	53.00 (25.00, 146.50)	107.00 (36.00, 865.00)	<0.001
Ast, M (Q₁, Q₃)	99.00 (43.00, 322.00)	51.50 (29.00, 132.75)	74.50 (38.00, 183.50)	198.00 (83.00, 805.00)	110.00 (49.00, 289.00)	241.00 (69.00, 2501.00)	<0.001
Bilirubin Total, M (Q₁, Q₃)	1.10 (0.60, 2.60)	0.70 (0.40, 1.40)	0.95 (0.50, 2.30)	1.40 (0.70, 2.50)	1.20 (0.60, 3.30)	2.10 (0.90, 5.30)	<0.001
Platelet, M (Q₁, Q₃)	183.00 (126.50, 254.00)	214.00 (165.00, 302.75)	185.00 (129.75, 259.75)	168.00 (121.00, 228.00)	177.00 (121.00, 245.00)	164.00 (118.00, 230.00)	<0.001
Wbc, M (Q₁, Q₃)	15.70 (11.05, 21.70)	14.10 (10.40, 19.38)	15.75 (11.30, 21.10)	17.30 (13.10, 23.90)	15.30 (10.50, 21.75)	16.60 (10.70, 23.20)	<0.001
Resp Rate, M (Q₁, Q₃)	30.00 (25.25, 34.00)	29.00 (25.00, 34.00)	30.00 (25.00, 35.00)	30.00 (25.50, 34.00)	30.00 (26.00, 35.00)	30.00 (27.00, 34.00)	0.343
Sbp Ni, M (Q₁, Q₃)	131.00 (117.00, 148.00)	133.50 (121.00, 153.75)	131.00 (116.00, 148.00)	128.00 (115.00, 147.00)	130.00 (117.00, 146.00)	133.00 (112.00, 147.00)	0.039
Dbp Ni, M (Q₁, Q₃)	81.00 (69.00, 96.00)	83.00 (73.00, 97.00)	82.00 (69.00, 96.00)	80.00 (67.00, 99.00)	81.00 (68.50, 95.00)	78.00 (68.00, 97.00)	0.605

Rbc, erythrocyte; ALT, alanine aminotransferase; AST, aspartate aminotransferase; WBC, leukocyte; Sbp, systolic blood pressure; Dbp, diastolic blood pressure.

#### 3.4.3 Comparison of the nursing process and outcomes

Median ICU length of stay (LOS) varied significantly across groups (overall median 12.52 days [IQR, 8.64–20.10]; “Low-stable group”: 12.18 [8.04–19.89], “low-slowly declining group”: 11.61 [8.29–18.69], “high-rapidly decline group”: 12.62 [9.26–20.99], “Moderate-slow declining group”: 13.67 [9.13–20.81], and “high-slow decline group”: 12.56 [8.10–21.32]; *p* = 0.025). Mortality at 28 days differed among groups (“Low-stable group”: 23.2%, “low-slowly declining group”: 32.1%, “high-rapidly decline group”: 25.8%, “Moderate-slow declining group”: 33.1%, “high-slow decline group”: 46.8%; *p* < 0.001). Dexmedetomidine use in the ICU also demonstrated group-wise variation (“Low-stable group”: 58.5%, “low-slowly declining group”: 57.3%, “high-rapidly decline group”: 52.4%, “Moderate-slow declining group”: 51.0%, “high-slow decline group”: 45.4%; *p* = 0.018). Total daily fluid intake varied widely among the groups (*p* < 0.001 for all). The use of vasopressors was similar in each group (*p* = 0.237). No statistically significant differences were observed in ICU utilization of propofol (*p* = 0.60), midazolam (*p* = 0.69), tramadol (*p* = 0.48), or vasoactive agents (*p* = 0.24; Table 5).

**Table 5:** Nursing process and outcomes were compared.

Variables	Total (*n* = 1,575)	Trajectory 1 (*n* = 246)	Trajectory 2 (*n* = 464)	Trajectory 3 (*n* = 225)	Trajectory 4 (*n* = 435)	Trajectory 5 (*n* = 205)	*p*
Los ICU, M (Q₁, Q₃)	12.52 (8.64, 20.10)	12.18 (8.04, 19.89)	11.61 (8.29, 18.69)	12.62 (9.26, 20.99)	13.67 (9.13, 0.81)	12.56 (8.10, 21.32)	0.025
Hosp Outcome 28d, n(%)							<0.001
No	1,071 (68.00)	189 (76.83)	315 (67.89)	167 (74.22)	291 (66.90)	109 (53.17)	
Yes	504 (32.00)	57 (23.17)	149 (32.11)	58 (25.78)	144 (33.10)	96 (46.83)	
Propofol ICU Used, n(%)							0.603
No	189 (12.00)	30 (12.20)	61 (13.15)	20 (8.89)	54 (12.41)	24 (11.71)	
Yes	1,386 (88.00)	216 (87.80)	403 (86.85)	205 (91.11)	381 (87.59)	181 (88.29)	
Dexmedetomidine ICU Used, n(%)							0.018
No	732 (46.48)	102 (41.46)	198 (42.67)	107 (47.56)	213 (48.97)	112 (54.63)	
Yes	843 (53.52)	144 (58.54)	266 (57.33)	118 (52.44)	222 (51.03)	93 (45.37)	
Midazolam ICU Used, n(%)							0.693
No	1,185 (75.24)	187 (76.02)	342 (73.71)	177 (78.67)	324 (74.48)	155 (75.61)	
Yes	390 (24.76)	59 (23.98)	122 (26.29)	48 (21.33)	111 (25.52)	50 (24.39)	
Tramadol ICU Used, n(%)							0.476
No	1,463 (92.89)	232 (94.31)	429 (92.46)	204 (90.67)	404 (92.87)	194 (94.63)	
Yes	112 (7.11)	14 (5.69)	35 (7.54)	21 (9.33)	31 (7.13)	11 (5.37)	
Vasoactive, n(%)							0.237
No	260 (16.51)	52 (21.14)	71 (15.30)	31 (13.78)	72 (16.55)	34 (16.59)	
Yes	1,315 (83.49)	194 (78.86)	393 (84.70)	194 (86.22)	363 (83.45)	171 (83.41)	
Liquid Input day 1, M (Q₁, Q₃)	19861.00 (11857.50, 29528.50)	13064.00 (7595.00, 19181.50)	17630.00 (10747.50, 23925.00)	28700.00 (19620.00, 37806.00)	20990.00 (12828.50, 31271.00)	25854.00 (15470.00, 35193.00)	<0.001
Liquid Input day 2, M (Q₁, Q₃)	14300.00 (9055.00, 21977.00)	9740.00 (6815.50, 14237.50)	13205.00 (8351.25, 19305.00)	15930.00 (10925.00, 23730.00)	15655.00 (10280.00, 23862.50)	19736.00 (13570.00, 27988.00)	<0.001
Liquid Input day 3, M (Q₁, Q₃)	12750.00 (8292.50, 19730.00)	9899.00 (6750.00, 13472.50)	11625.00 (7997.50, 17551.25)	13420.00 (8820.00, 20000.00)	13368.00 (9285.00, 20755.00)	18496.00 (12800.00, 27285.00)	<0.001
Liquid Input day 4, M (Q₁, Q₃)	11890.00 (7300.00, 18791.50)	10250.00 (5992.40, 14600.00)	10795.00 (7117.50, 17344.00)	10970.00 (7320.00, 18100.00)	12404.00 (7570.00, 19530.00)	17900.00 (10360.00, 24600.00)	<0.001
Liquid Input day 5, M (Q₁, Q₃)	0970.00 (6385.00, 18690.00)	9205.00 (5800.00, 15412.50)	9872.50 (5859.75, 15901.50)	10680.00 (6240.00, 18320.00)	10970.00 (6600.00, 18910.00)	17422.00 (10470.00, 23577.00)	<0.001

### 3.5 Survival analysis affecting survival time

**Figure 3:**
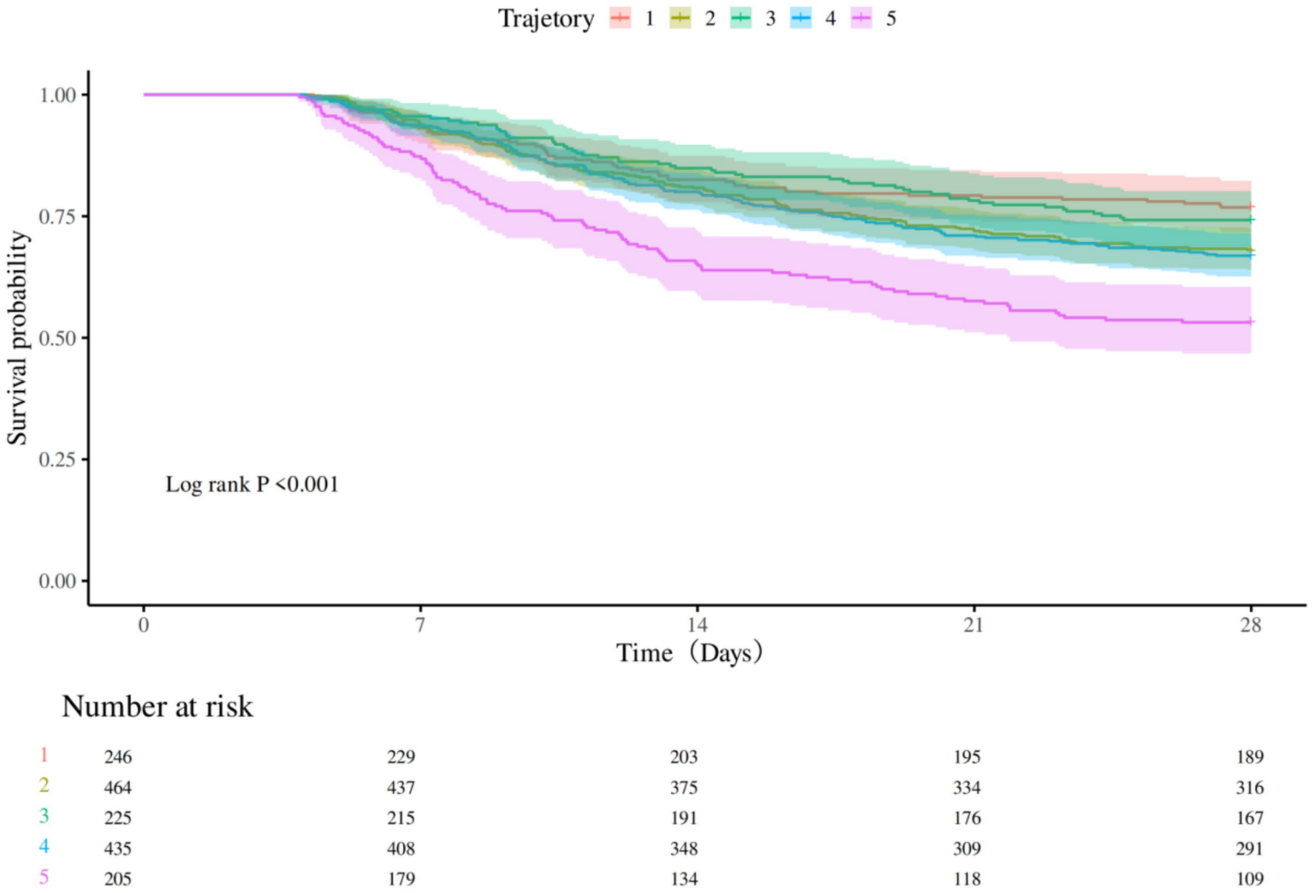
Kaplan–Meier survival curve by the group.

Kaplan–Meier survival analysis was used to construct survival curves for patients with the five phenotypes of sepsis. The abscissa is the observation time, and the ordinate is the survival status of patients 28 days after admission (survival rate). Patients in trajectory 5 had the highest 28-day mortality, and patients in trajectory 3 had the lowest 28-day mortality, with a significant difference in 28-day mortality between trajectory groups (*p* < 0.0001; Figure 3). Next, we performed univariate Cox regression analysis with survival time and survival outcome as dependent variables and other factors as covariates, and the results revealed that Sofa score, APSIII, bilirubin total, RBC, WBC, hemoglobin, propofol, dexmedetomidine, tramadol, congestive heart failure, and metastatic solid tumor were the influencing factors for the survival time of patients. According to the results of univariate analysis, statistically significant factors were included in the multivariate Cox regression analysis. The results revealed that APSIII, WBC, dexmedetomidine, tramadol, congestive heart failure, and metastatic solid tumor were the influencing factors affecting the survival time of patients (Table 6).

**Table 6:** Results from the univariate and multivariate Cox proportional hazard models.

Variables	Univariate					Multivariate				
	*β*	S. E	Z	*p*	HR (95%CI)	*β*	S. E	Z	*p*	HR (95%CI)
Gender										
F					1.00 (Reference)					
M	−0.10	0.09	−1.07	0.284	0.91 (0.76–1.08)					
Admission Age	−0.00	19.39	−0.00	1.000	1.00 (0.00–32030507569812488.00)					
Sofa Score	0.04	0.01	3.03	0.002	1.04 (1.02–1.07)	0.03	0.02	1.58	0.113	1.03 (0.99–1.06)
APSIII	0.01	0.00	6.50	<0.001	1.01 (1.01–1.01)	0.01	0.00	3.67	<0.001	1.01 (1.01–1.01)
Creatinine	0.02	0.02	0.81	0.416	1.02 (0.97–1.06)					
Alt	−0.00	0.00	−0.96	0.336	1.00 (1.00–1.00)					
Ast	−0.00	0.00	−0.88	0.379	1.00 (1.00–1.00)					
Bilirubin total	0.01	0.01	2.89	0.004	1.01 (1.01–1.03)	0.00	0.01	0.23	0.821	1.00 (0.99–1.01)
RBC	−0.15	0.06	−2.65	0.008	0.86 (0.77–0.96)	−0.10	0.14	−0.76	0.450	0.90 (0.69–1.18)
WBC	0.01	0.00	2.99	0.003	1.01 (1.01–1.01)	0.01	0.00	2.37	0.018	1.01 (1.01–1.01)
Platelet	0.00	0.00	0.70	0.482	1.00 (1.00–1.00)					
Hemoglobin	−0.05	0.02	−2.41	0.016	0.95 (0.92–0.99)	0.01	0.05	0.12	0.903	1.01 (0.92–1.11)
Heart Rate	−0.00	0.00	−0.05	0.960	1.00 (1.00–1.00)					
Resp Rate	0.01	0.01	1.80	0.072	1.01 (1.00–1.02)					
Sbp	0.00	0.00	1.67	0.094	1.00 (1.00–1.01)					
Dbp	0.00	0.00	0.59	0.557	1.00 (1.00–1.01)					
Propofol ICU Used										
No					1.00 (Reference)					1.00 (Reference)
Yes	−0.37	0.12	−2.99	0.003	0.69 (0.54–0.88)	−0.04	0.13	−0.28	0.782	0.96 (0.75–1.25)
Dexmedetomidine ICU used										
No					1.00 (Reference)					1.00 (Reference)
Yes	−0.56	0.09	−6.25	<0.001	0.57 (0.48–0.68)	−0.49	0.09	−5.27	<0.001	0.61 (0.51–0.73)
Midazolam ICU used										
No					1.00 (Reference)					
Yes	−0.11	0.10	−1.03	0.302	0.90 (0.73–1.10)					
Tramadol ICU used										
No					1.00 (Reference)					1.00 (Reference)
Yes	−1.62	0.34	−4.80	<0.001	0.20 (0.10–0.38)	−1.58	0.34	−4.66	<0.001	0.21 (0.11–0.40)
Congestive Heart Failure										
No					1.00 (Reference)					1.00 (Reference)
Yes	0.19	0.09	2.10	0.035	1.21 (1.01–1.45)	0.33	0.10	3.46	<0.001	1.39 (1.16–1.68)
Cerebrovascular Disease										
No					1.00 (Reference)					
Yes	0.10	0.12	0.86	0.390	1.11 (0.88–1.40)					
Chronic Pulmonary Disease										
No					1.00 (Reference)					
Yes	0.10	0.10	0.97	0.331	1.10 (0.91–1.33)					
Diabetes										
No					1.00 (Reference)					
Yes	−0.05	0.10	−0.48	0.632	0.95 (0.79–1.15)					
Renal Disease										
No					1.00 (Reference)					
Yes	0.12	0.10	1.20	0.231	1.13 (0.93–1.38)					
Malignant Cancer										
No					1.00 (Reference)					
Yes	0.14	0.14	1.02	0.307	1.15 (0.88–1.51)					
Metastatic Solid Tumor										
No					1.00 (Reference)					1.00 (Reference)
Yes	0.83	0.18	4.65	<0.001	2.28 (1.61–3.23)	0.71	0.18	3.90	<0.001	2.03 (1.42–2.90)
Vasoactive										
No					1.00 (Reference)					
Yes	0.25	0.13	1.93	0.054	1.28 (1.00–1.65)					

Lactate levels are not only affected by the severity of sepsis, but also by many other factors such as the treatment regimen used, liver function, and so on. To exclude the influence of these confounding variables, we used the multi-model method to control the confounding factors, and adjusted the confounding factors with a *p*-value of < 0.05 selected in the univariate analysis (Table 7). The results showed that lactate was an independent predictor of death in patients with sepsis, and LAC1 might be due to the fact that early lactate was more affected by therapeutic intervention or underlying diseases, or there might be unmeasured confounding factors that affected the results.

**Table 7:** Association between lactate levels and the risk of 28-day mortality in patients with sepsis.

Variables	Model 1		Model 2	
	HR (95%CI)	*p*	HR (95%CI)	*p*
LCA1	1.02 (1.00–1.04)	0.109	1.00 (0.97–1.02)	0.889
LAC2	1.07 (1.04–1.09)	<0.001	1.04 (1.01–1.07)	0.006
LAC3	1.09 (1.06–1.13)	<0.001	1.06 (1.03–1.10)	<0.001
LAC4	1.14 (1.10–1.17)	<0.001	1.11 (1.07–1.15)	<0.001
LAC5	1.19 (1.16–1.22)	<0.001	1.19 (1.15–1.22)	<0.001

Model 1: Crude. Model 2: Adjust: propofol, dexmedetomidine, tramadol, congestive heart failure, metastatic solid tumor, APSIII, sofa, bilirubin total, RBC, WBC, and hemoglobin.

## 4 Discussion

We report on a method for identifying subphenotypes of sepsis patients based on lactate trajectories. Through group-based trajectory modeling, we identified and validated five groups of sepsis patients with different lactate trajectories, namely, “Low-stable group,” “low-slowly declining group,” “high-rapidly decline group,” “Moderate-slow declining group,” and “high-slow decline group.” We also explored the relationships between different lactate trajectories and 28-day mortality in sepsis patients. Among them, the “Low-stable group” had the lowest 28-day mortality rates. Patients with a “high-slow decline group” had the highest 28-day mortality. We also used Cox univariate and multivariate analyses to identify the factors affecting survival time, and the results revealed that APSIII, WBC, dexmedetomidine, tramadol, congestive heart failure, and metastatic solid tumor were the influencing factors affecting survival time. In addition, we compared the demographic and complication characteristics, physiological characteristics, and nursing process of the five subphenotypes.

The study found that patients in the “high-rapidly decline group” and “high-slow decline group” required substantially greater fluid resuscitation volumes during the initial 24 h compared to other cohorts (*p* < 0.001), though no significant intergroup differences were observed in vasopressor utilization rates (*p* = 0.743). These findings corroborate current resuscitation guidelines (13), emphasizing that protocolized fluid administration remains fundamental for reversing sepsis-induced tissue hypoperfusion and achieving lactate clearance targets. Meanwhile, early and protocolized administration of vasoactive agents is associated with significant mortality reduction in sepsis and septic shock (14). These findings are highly important for understanding the heterogeneity of sepsis patients and may determine differences for future studies to provide information on the phenotypic response to treatment.

High lactic acidosis is a symbolic feature (15, 16) of shock, and the degree of elevated lactic acid concentration is directly related (17) to the severity of the shock status and mortality. In this study, we also confirmed that the blood lactic acid level is related to the severity of sepsis or multiple organ failure and that the level of useful markers of blood lactic acid is high, resulting in increased mortality (7). This has to do with Haoyue Zhang (18), etc. This is consistent with the research results of Haoyue Zhang (18).

At present, the research on sepsis subtypes is still in the preliminary stage, and there is no unified standard for inclusion indicators and research methods. These different subtypes have their own characteristics (19) in terms of classification, data sources, prognostic indicators, and treatment heterogeneity. For example, a prospective cohort study by Davenport et al. (20) from the United Kingdom in 2016 identified two sepsis subtypes via the cluster method by detecting whole gene expression in peripheral blood leukocytes. The sepsis response signatures (SRSs) are type 1 (108 cases, 41%) and type 2; type 1 SRSs are more severe and more prone to hypotension and the use of vasoactive drugs. The 14-day, 28-day, and 6-month mortality rates of SRS 1 patients were significantly higher than those of SRS 2 patients. By including 540 patients with sepsis-induced ARDS in 2018, Professor Calfee’s (21) team established two subtypes on the basis of cluster analysis of clinical indicators and biomarkers. Patients with type 1 disease have a greater inflammatory response, worse prognosis, and higher 28-day mortality. Seymour et al. (22) used cluster statistics and machine learning, and simulation methods to retrospectively analyze data according to clinical big data and machine learning methods. Four phenotypes (*α*, *β*, *γ*, and *δ*) were identified based on 29 early clinical indicators in more than 20,000 sepsis patients, and the above phenotypes were validated in more than 40,000 sepsis patients: one previous cohort study and three previous RCTs. The results revealed that the four sepsis phenotypes presented different clinical characteristics. The α phenotype was the most common, and the dose of vasopressor drugs was the lowest. Patients with the β phenotype were older and had more complications and renal insufficiency. Patients with the γ phenotype had a greater inflammatory response and respiratory dysfunction. Liver dysfunction and shock are more common in patients with the δ phenotype. The 28-day and 365-day mortality rates of the four phenotypes were significantly different, and the levels of inflammatory markers were also significantly different.

This study highlights that distinct clinical phenotypes necessitate differentiated treatment strategies. Personalized therapeutic regimens, tailored to specific patient phenotypes, may enhance treatment efficacy while mitigating adverse effects. For example, patients in the high-slow decline group may require intensified interventions targeting infection management, blood transfusion support, and hepatic/renal function preservation, alongside high-intensity monitoring to promptly identify complications or disease progression. Conversely, low-risk phenotypes (e.g., low-stable group) could benefit from reduced monitoring frequency to minimize unnecessary resource utilization. By stratifying patients into risk-based phenotypes, healthcare systems can optimize resource allocation, prioritizing critical care for high-need populations.

Of course, our results still have certain limitations. First, this study was a single-center retrospective cohort study. Although the effects of many confounders were considered, there may still be some potential confounders (e.g., interventions and variations in ICU protocols) influencing the results, and these results are hypothesis-generating and need to be validated by multicenter prospective studies. Second, this study utilized electronically recorded health data, but the use of these data has limitations such as document bias, missing data, variability in measurement frequency, and unobserved confounding factors. These problems may affect the accuracy and extrapolability of the results. Third, this study focused on the prognostic value of lactate dynamics during the acute phase of sepsis (within 5 days of admission) and failed to assess lactate fluctuations associated with late complications such as secondary infection or late organ failure. Future prospective studies need to extend the monitoring window to fully evaluate the role of lactate in the full course of sepsis management. Finally, relying only on repeated measurements of lactate for patient risk stratification has some lag, so it needs to be combined with other measures to improve accuracy.

## 5 Conclusion

In this study, we successfully identified different subtypes of sepsis by analyzing lactate trajectories. Combined with the dynamic changes in late levels, the GBTM model was adopted to stratify sepsis risk. The clinical application of this model was able to significantly shorten the time of patient assessment, allowing providers to tailor treatment and care programs to patients on the basis of the predicted results and to implement early interventions. This can not only help reduce the mortality rate of patients but also improve the clinical prognosis of patients and achieve better treatment results.

## Data Availability

The original contributions presented in the study are included in the article/supplementary material, further inquiries can be directed to the corresponding author.
